# Comorbid Substance Use in Schizophrenia: Multivariate Insight into Symptoms, Functioning, and Premorbid Adjustment

**DOI:** 10.1192/j.eurpsy.2026.10219

**Published:** 2026-07-31

**Authors:** E. Caporusso, P. Pezzella, A. Perrottelli, L. Giuliani, A. Melillo, F. F. Marzocchi, M. Amore, P. Rocca, A. Rossi, A. Bertolino, G. M. Giordano, A. M. Mucci, S. Galderisi, M. Maj

**Affiliations:** 1 Department of Pscyhiatry, University of Campania “Luigi Vanvitelli”, Naples; 2 Department of Neurosciences, Rehabilitation, Ophthalmology, Genetics and Maternal and Child Health, Section of Psychiatry, University of Genoa, Genova; 3 Department of Neuroscience, University of Turin, Turin; 4 Department of Biotechnological and Applied Clinical Sciences, Section of Psychiatry, University of L’Aquila, L’Aquila; 5 Department of Basic Medical Science, Neuroscience and Sense Organs, University of Bari ‘Aldo Moro’, Bari, Italy

## Abstract

**Introduction:**

Substance use disorder (SUD) is highly prevalent in individuals with schizophrenia, with a lifetime prevalence of around 40%, and is consistently associated with poor outcomes. However, prior studies have yielded inconsistent results regarding its associations with negative symptoms, cognition, and functioning. The present study adopted a multivariate approach to delineate the clinical and functional profile of patients with schizophrenia and comorbid SUD.

**Objectives:**

We aimed to identify sociodemographic, clinical, premorbid, cognitive, functional, and coping dimensions associated with SUD in schizophrenia, using data from a large multicenter cohort.

**Methods:**

This cross-sectional study included 922 clinically stable patients with DSM-5 schizophrenia consecutively recruited within the Italian Network for Research on Psychoses. SUD diagnosis was established with the MINI. Assessments included PANSS, BNSS, PAS, SLOF, MCCB, MSCEIT, and Brief COPE. Group comparisons were followed by multivariate logistic regression and causal mediation analysis.

**Results:**

Of 922 participants, 240 (26.0%) had comorbid SUD. Compared to non-SUD, they were more often male (80.8% vs 65.5%), younger (38.4 vs 40.8 years), with earlier onset (22.5 vs 24.6 years) and more hospitalizations (5.1 vs 3.2). In the SUD group, PAS showed poorer academic functioning (p < .00001) with relatively better social functioning (p < .00001) across childhood and adolescence. BNSS scores were lower for both MAP (11.5 vs 13.3, p = .003) and EXP domains (19.5 vs 21.1, p = .033). No group differences emerged for PANSS positive, disorganization, cognition, or social cognition. Regression indicated higher odds of SUD with maladaptive coping (COPE substance use OR 1.44, p < .001; self-distraction OR 1.21, p < .001), poorer PAS academic functioning in early adolescence (OR 1.44, p = .003), and better SLOF physical functioning (OR 1.18, p = .030). Protective effects were observed for PAS social functioning (OR 0.55, p < .001), higher BNSS EXP scores (OR 0.79, p = .011), and better SLOF work skills (OR 0.96, p = .016). Mediation analysis showed that the protective effect of premorbid social functioning was largely direct, with only about 10% mediated by lower severity of EXP symptoms, suggesting that reduced expressive deficits may indirectly increase opportunities for substance exposure.

**Conclusions:**

In our study, comorbid SUD in schizophrenia emerged as a distinct clinical profile, characterized by early academic difficulties, preserved premorbid social functioning, lower severity of negative symptoms, and maladaptive coping strategies. These findings suggest that SUD may reflect a different trajectory of illness, rather than its greater severity. Preventive school-based initiatives, together with structured psychosocial interventions, could play a crucial role in reducing vulnerability in this subgroup.

**Disclosure of Interest:**

None Declared

